# Integrated Proteomics and Metabolomics Analyses Reveal That Phosphatidylethanolamine Reprograms Macrophage Immunometabolism and Attenuates LPS-Driven Inflammation

**DOI:** 10.1021/acs.jproteome.5c01131

**Published:** 2026-04-08

**Authors:** Tatiana Maurício, Bruno Neves, M. Rosário Domingues, Pedro Domingues

**Affiliations:** † Mass Spectrometry Centre, LAQV-REQUIMTE, Department of Chemistry, 56062 University of Aveiro, Santiago University Campus, Aveiro 3810-193, Portugal; ‡ CESAM - Centre for Environmental and Marine Studies, Department of Chemistry, University of Aveiro, Santiago University Campus, Aveiro 3810-193, Portugal; § Department of Medical Sciences and Institute of Biomedicine, iBiMED, University of Aveiro, Aveiro 3810-193, Portugal

**Keywords:** phosphatidylethanolamine, macrophages, lipopolysaccharide, immunometabolism, proteomics, metabolomics, oxidative stress, inflammation

## Abstract

Phospholipids are key regulators of immune metabolism, yet their specific influence on macrophage function remains incompletely defined. We investigated how phosphatidylethanolamine (PE) species with distinct acyl chains (PE18:0/22:6 and PE18:0/20:4) modulate RAW264.7 macrophages under resting and LPS-stimulated conditions using LC-MS/MS-based proteomics and metabolomics, followed by qPCR validation. LPS elicited a robust M1-like phenotype with strong upregulation of Ptgs2, Nos2, Nfkb1, and Nfkb2. PE supplementation alone did not induce a classical pro-inflammatory profile but significantly remodeled protein expression, enhancing antioxidant defenses, including catalase, Hmox1 and Prdx1. In the context of LPS activation, PE selectively attenuated inflammatory signaling by downregulating Nfkb1, Nfkb2, and Ptgs2 while further enhancing proteins linked to oxidative stress response (Prdx1 and Hmox1) and lipid metabolism (CD36 and Abcc1). qPCR corroborated these effects: both PE species reduced LPS-induced *Il1b* and *Ptgs2* mRNA levels while increasing *Prdx1*, *Hmox1*, and *Cd36* transcription. Metabolomics converged with these findings, indicating reinforced glutathione metabolism and context-dependent shifts in purine and amino-acid pathways consistent with a restrained inflammatory phenotype. Collectively, native PE species reprogram macrophage immunometabolism, mitigating LPS-driven inflammation while strengthening Nrf2-mediated antioxidant and immune-supportive pathways.

## Graphical abstract

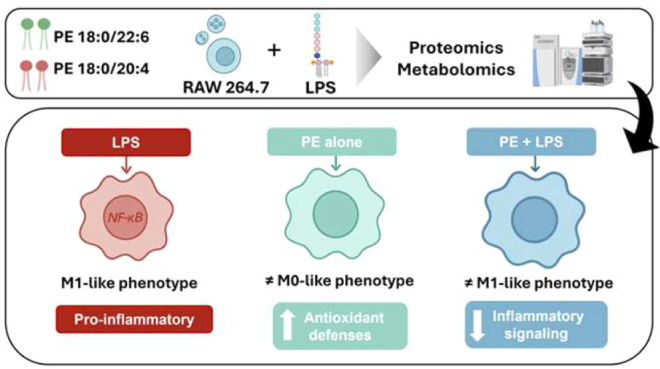

## 1 Introduction

Macrophages are highly adaptable immune cells that play central roles in host defense, tissue homeostasis, and the regulation of inflammation. Their functional plasticity enables rapid reprogramming in response to environmental signals, allowing them to adopt distinct phenotypes that support different phases of the immune response. Traditionally, macrophages are classified into M1 (pro-inflammatory) and M2 (anti-inflammatory or pro-resolving) subsets. These categories, largely derived from *in vitro* models, describe polarization driven by LPS or IFN-γ for M1-like activation, and by interleukin (IL)-4 or IL-13 for M2-like activation.^1–3^

Recent studies have shown that macrophage subsets, particularly M1 and M2, display distinct metabolic signatures.^4^ M1 macrophages primarily rely on glycolysis and fatty acid synthesis to sustain pro-inflammatory activity,^4,5^ whereas M2 macrophages, associated with tissue repair and inflammation resolution, depend mainly on oxidative phosphorylation (OXPHOS) and fatty acid oxidation (FAO).^6^ This metabolic reprogramming directly influences macrophage functions, including cytokine production, phagocytosis, and antigen presentation. These adaptations are reflected in their proteomic and lipidomic profiles, as changes in protein expression, signaling pathways, and lipid composition correspond to specific activation states.^7,8^

Lipid metabolism plays a crucial role in macrophage polarization and function.^9,10^ Among the various lipid classes, phospholipids (PL) have attracted increasing attention due to their dual role as structural components of cellular membranes and as active modulators of cell signaling. These regulatory functions include modulation of cytokine production, alteration of gene expression, regulation of inflammatory metabolic pathways, and attenuation of oxidative stress. While the immunomodulatory effects of phosphatidylcholine (PC) on macrophages have been explored,^11,12^ the specific role of phosphatidylethanolamine (PE) in their immunometabolism remains poorly explained.

PE, the second most abundant PL in mammalian membranes, is essential for maintaining membrane integrity, supporting mitochondrial function, and regulating oxidative phosphorylation.^6,13,14^ It has been reported to exert anti-inflammatory effects on macrophages through multiple mechanisms. PE attenuates oxidized low-density lipoprotein (OX-LDL)-induced inflammation by reducing pro-inflammatory cytokine expression and inhibiting NLRP1 inflammasome activation.^15^ Furthermore, PE suppresses palmitic acid (PA)-induced macrophage inflammatory status,^16^ downregulating NLRP3 inflammasome expression while upregulating suppressor of cytokine signaling 3 (SOCS3), a critical negative regulator of inflammation. In addition, eicosapentaenoate (EPA)-PE was shown to inhibit NF-κB activation in RAW 264.7 macrophages.^17^

Despite these insights, the mechanisms underlying PE’s immunomodulatory effects remain incompletely understood. It is unclear whether these effects are primarily driven by the ethanolamine headgroup or also influenced by the esterified fatty acid moiety. Beyond headgroup chemistry, the central question is whether native PE species help shape membrane organization and lipid-mediator availability, thereby influencing macrophage redox and metabolism in a context-dependent manner (resting vs LPS). To address this gap, we investigated the effects of two specific PE species containing polyunsaturated fatty acids (PUFA), PE18:0/22:6 (containing docosahexaenoic acid, DHA) and PE18:0/20:4 (containing arachidonic acid, AA), on RAW 264.7 macrophages. This study aimed to elucidate how these distinct PE species reprogram macrophage immunometabolism and function through proteomic and metabolomic profiling under both basal and LPS-induced inflammatory conditions. By identifying proteomic and metabolomic signatures associated with PE supplementation, we sought to clarify their roles in modulating inflammatory signaling and oxidative stress responses.

## 2 Materials and Methods

### 2.1 Chemicals and Reagents

1-Stearoyl-2-arachidonoyl-*sn*-glycero-3-phosphoethanolamine (PE18:0/20:4) and 1-stearoyl-2-docosahexaenoyl-*sn*-glycero-3-phosphoethanolamine (PE18:0/22:6) were obtained from Avanti Polar Lipids (Alabaster, AL, USA). HPLC-grade solvents and Milli-Q water (0.22 μm filtered) were used throughout. Dulbecco’s Modified Eagle Medium (DMEM), LPS (*E. coli* 055:B5), and fetal bovine serum (FBS) were from Sigma-Aldrich (St. Louis, MO, USA). TRIzol reagent was from Invitrogen (Barcelona, Spain), and NZY First-Strand cDNA Synthesis Kit and NZYSupreme qPCR Green Master Mix were from NZYtech (Lisbon, Portugal).

### 2.2 Phospholipid Vesicles Preparation

PE18:0/22:6 and PE18:0/20:4 vesicles were prepared by the thin-film hydration method.^18^ Phospholipids dissolved in chloroform were evaporated under nitrogen to form a dry film, which was hydrated with DMEM to the desired volume. Vesicle size was homogenized by repeated vortex-sonication cycles in an ultrasonic water bath. Phospholipid vesicles were stored at −20 °C overnight prior to cell treatment.

### 2.3 Cell Culture and Treatment

RAW 264.7 macrophages (ATCC TIB-71) were cultured in high-glucose DMEM supplemented with 10% FBS, 100 μg/mL streptomycin, 100 U/mL penicillin, and 1.5 g/L sodium bicarbonate, at 37 °C and 5% CO_2_. Cells were subcultured every 2–3 days to maintain 70–80% confluency, routinely tested for mycoplasma contamination, and used at passages below 30.

#### 2.3.1 Cell Viability and Nitric Oxide Measurement

Cell viability and nitric oxide (NO) production were assessed in preliminary assays using the resazurin reduction and Griess reaction methods, respectively, as previously described,^19^ to determine optimal conditions for PE18:0/22:6 and PE18:0/20:4 treatments.

#### 2.3.2 PE Supplementation and LPS Stimulation

Macrophages were seeded at 2 × 10^6^ cells/well in 6-well plates and incubated overnight. Cells were treated with 100 μM PE18:0/22:6 or PE18:0/20:4 vesicles for 2 h, followed by stimulation with 100 ng/mL LPS where indicated. The PE concentration (100 μM) was selected based on prior work showing no loss of RAW264.7 viability at this dose and dose-dependent inhibition of LPS-induced NO production.^19^ After 24 h, six experimental conditions were obtained: untreated control (CT); LPS-treated macrophages (CT_LPS; 100 ng/mL); PE18:0/22:6; PE18:0/20:4; PE18:0/22:6_LPS, and PE18:0/20:4_LPS (*n* = 6 per group). Cells were washed with cold PBS, collected, and stored at −80 °C for further analysis.

### 2.4 Protein and Metabolite Extraction

Proteins were isolated using a modified Simplex extraction method.^20^ Cell pellets were lysed in methanol (MeOH) via three cycles of freezing in liquid nitrogen, thawing, and sonication in a cold ultrasonic water-bath, with vortex intercalated between cycles. Lipids were then extracted by adding methyl *tert*-butyl ether (MTBE) and incubating the samples at 4 °C for 1 h under continuous shaking.

Phase separation was induced with 188 μL 0.1% ammonium acetate, followed by centrifugation (10,000 g, 10 min, 4 °C). The upper lipid phase was removed, and proteins were precipitated from the lower phase by adding MeOH (4:1, v/v), incubated at −20 °C for 2 h, and centrifuged (13,000 g, 12 min, 4 °C). Metabolite-containing supernatants were collected, dried in a SpeedVac concentrator and stored at −80 °C. The protein pellet was resuspended in a 1:4 (v/v) mixture of 8 M urea and 50 mM ammonium bicarbonate, and quantified by RC/DC assay (Bio-Rad, Hercules, CA, USA).

### 2.5 Protein Digestion and Peptide Desalting

Protein digestion was performed using an adapted in-solution tryptic digestion protocol (Thermo Fisher Scientific). Samples (20 μg protein) were reduced with 50 mM dithiothreitol (DTT) (56 °C, 45 min), alkylated with 15 mM iodoacetamide (RT, 30 min, dark), and quenched with 10 mM DTT (RT, 15 min). Urea concentration was diluted to <1 M with 50 mM ammonium bicarbonate before overnight digestion with trypsin (1:50, w/w) at 37 °C. Digests were centrifuged (2,500 g, 10 min), and supernatants were desalted using Pierce C18 Spin Columns (Thermo Fisher Scientific). Peptides were dried in a SpeedVac and reconstituted in 0.1% formic acid for LC-MS analysis.

### 2.6 Proteome Analysis by LC-MS/MS

Tryptic peptide digests were reconstituted in 40 μL of 0.1% formic acid in LC-MS-grade water and analyzed on a Q-Exactive hybrid quadrupole-Orbitrap mass spectrometer (Thermo Fisher Scientific, Bremen, Germany) coupled to an Ultimate 3000 Dionex nanoflow HPLC system. Peptides were separated on an EASY-Spray C18 column (75 μm × 150 mm, 2 μm, 100 Å) at 35 °C using a linear gradient of 5–24% buffer B (80% acetonitrile, 0.1% formic acid) over 50 min, followed by 24–36% B in 10 min and held for 5 min at 300 nL min^–1^. The instrument operated in positive ion mode (2.0 kV, 250 °C capillary temperature). Full MS scans were acquired at 70,000 resolution (AGC 1 × 10^6^, IT 100 ms), and the 10 most intense ions were fragmented by higher-energy collisional dissociation (resolution 17,500; AGC 5 × 10^4^; IT 50 ms; CE 28; isolation width 1.2 Th; dynamic exclusion 30 s).

Raw data were processed in Proteome Discoverer v2.2 using SEQUEST HT and MS Amanda 2.0 with Percolator validation against the *Mus musculus* SwissProt database (accessed June 2024). Search parameters included carbamidomethylation of Cys (fixed), oxidation of Met and N-terminal acetylation (variable), 10 ppm precursor and 0.02 Da fragment tolerances, and up to two missed cleavages. A 1% false discovery rate (FDR) was applied at the peptide and protein levels, and only proteins with ≥2 unique peptides (≥6 aa) were retained. Proteins exhibiting low variability were excluded prior to analysis. Specifically, proteins with low-variance features (interquartile range < 0.5) and low-abundance (mean log_2_ relative abundance < 1) were removed.

### 2.7 Metabolomics Analysis by LC-MS/MS

Samples for metabolomics analysis were resuspended in 80% LC-MS-grade methanol containing the internal standard Leu-Tyr (0.02 mg/mL; Sigma-Aldrich, St. Louis, MO, USA). Metabolite profiling was performed by HILIC-LC-MS/MS using the same instrumental configuration described previously.^11^ Mobile phase A consisted of ACN:H_2_O (95:5, v/v) and mobile phase B of ACN:H_2_O (50:50, v/v), both containing 10 mM ammonium formate and 0.1% formic acid. The gradient started with 100% B for 1 min, followed by 0–50% A over 15 min, and was held for 5 min. The organic lipid fraction was previously analyzed by C18-HPLC-MS/MS for oxidized PE (oxPE) species, including PE18:0/20:4;O and PE18:0/22:6;O.^19^

The mass spectrometer operated in both positive (3.1 kV) and negative (−2.8 kV) ion modes (capillary temperature 360 °C; sheath gas flow 35 U). Full MS scans were acquired at resolution 70,000 (AGC 1 × 10^6^; IT 50 ms) over an *m*/*z* range of 65–900. In MS/MS runs, the 10 most intense ions were fragmented by HCD (resolution 17,500; AGC 1 × 10^3^; IT 50 ms; CE 20, 30, and 40; isolation width 1.5 Th; dynamic exclusion 30 s). Pooled quality control (QC) samples were prepared by combining equal volumes of each sample. This pooled QC was injected at regular intervals using the same analytical workflow as the study samples to monitor instrument stability throughout the run.

Raw data were processed in Compound Discoverer v3.3 (Thermo Fisher Scientific) using an integrated workflow including peak detection, alignment, normalization, and noise filtering. Metabolite identification was performed by spectral library matching against mzCloud and ChemSpider databases, and annotations were accepted only when supported by high-confidence MS/MS spectral similarity scores.

### 2.8 RNA Extraction and Quantitative PCR Analysis

RAW 264.7 cells were seeded at 1 × 10^6^ cells/well in 12-well plates (1 mL final volume) and treated for 24 h with PE18:0/22:6 or PE18:0/20:4 (100 μM). LPS (100 ng/mL) was added 2 h after PE supplementation. Total RNA was extracted using TRIzol reagent following the manufacturer’s instructions and stored at −80 °C in RNA Storage Solution (Ambion, Foster City, CA, USA). RNA concentration was determined by OD_260_ using a NanoDrop spectrophotometer (Wilmington, DE, USA), and 2 μg of total RNA were reverse-transcribed with the NZY First-Strand cDNA Synthesis Kit.

Quantitative PCR (qPCR) was performed in duplicate on a Bio-Rad CFX Connect system using 25 ng cDNA and SYBR Green chemistry. Each sample was analyzed in duplicate under the following cycling conditions: an initial denaturation at 95 °C for 2 min, followed by 40 cycles of 95 °C for 5 s, 55 °C for 10 s, and 72 °C for 10 s. Gene expression was analyzed with GenEx v7 (MultiD Analyses AB, Gothenburg, Sweden). *Hprt1* was selected as the primary reference gene based on prior stability assessment in RAW264.7 cells,^19,21^ using the geNorm and NormFinder algorithms. Primers were designed with Beacon Designer v7.2 (Premier Biosoft International) and validated prior to use (primer sequences are listed in Supplementary Table 1).

### 2.9 Statistical Analysis

Multivariate and univariate analyses were conducted in R v4.4.2 using RStudio 2025.09.0+387.^22^ Proteomic data were normalized by total sum, log-transformed, and analyzed by principal component analysis (PCA) with the FactoMineR^23^ and factoextra^24^ packages. Statistical significance across six experimental conditions was assessed by one-way ANOVA using rstatix, after confirming that the data was not heavily skewed and did not contain significant outliers,^25^ followed by Tukey’s posthoc test. *p*-values were adjusted for multiple testing using the Benjamini–Hochberg method (FDR cutoff = 0.05). Data visualization was performed in ggplot2;^26^ heatmaps were generated with pheatmap^27^ using Euclidean distance and the Ward.D clustering method.^28^ Gene Ontology (GO) enrichment analysis was performed with clusterProfiler,^29^ using UniProt annotations.^30^ Differentially abundant proteins were defined by *p* < 0.05 and |fold change| > 1. Enrichment significance was evaluated with FDR < 0.05, and results were visualized in ggplot2.

Metabolomics data were normalized by total sum, log_2_-transformed, and further normalized using an EigenMS-like singular value decomposition (SVD) approach.^31^ Visualizations were generated in ggplot2, and pathway analysis was performed in MetaboAnalyst 6.0 (http://www.metaboanalyst.ca/). For pairwise comparisons, metabolites identified as significant by Tukey’s posthoc test were imported into MetaboAnalyst. Pathway analysis parameters included: enrichment method: Hypergeometric Test; topology analysis: relative betweenness centrality; and organism-specific library: *Mus musculus* (KEGG). Pathways were considered significant with an impact value ≥ 0.2 and −log_10_(p) ≥ 2, applying FDR correction with a cutoff of 0.05 to ensure biological and statistical relevance.

## 3 Results

### 3.1 Global Proteomic Remodeling Induced by PE Supplementation

To elucidate the immunomodulatory effects of PE18:0/22:6 and PE18:0/20:4 on resting (M0-like) and LPS-activated (M1-like) macrophages, we profiled by nano-LC-MS the proteome of RAW 264.7 cells 24 h poststimulation. A total of 2,554 proteins were identified and semiquantified, of which 1,311 exhibited significant differential abundance (*p.adj* < 0.05) across the six experimental conditions.

PCA revealed extensive reorganization of the macrophage proteome (Figure 1). The first component (54.2% variance) separated LPS-treated from non-LPS groups, reflecting the dominant metabolic and inflammatory effect of LPS. The second component (12.2% distinguished control groups (CT and CT_LPS) from PE-supplemented macrophages, indicating additional remodeling independent of LPS. Both PE18:0/22:6 and PE18:0/20:4 induced distinct yet overlapping proteomic signatures, suggesting shared mechanisms regardless of acyl-chain composition. Under LPS stimulation, PE18:0/22:6_LPS and PE18:0/20:4_LPS also clustered together, demonstrating a consistent adaptive response to combined PE and LPS exposure. Overall, PE supplementation markedly reprogrammed the macrophage proteome under both basal and inflammatory conditions.

**Figure 1:**
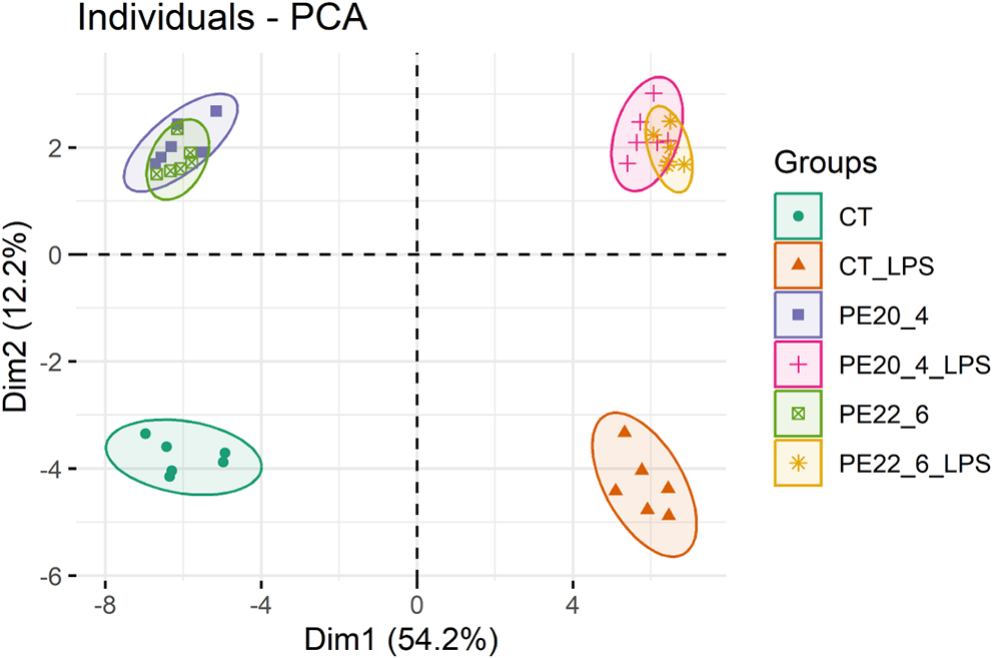
Principal component analysis (PCA) score plot of the proteomic data set showing sample distribution across the six experimental conditions: control (CT), LPS-treated control (CT_LPS), PE18:0/20:4, PE18:0/20:4_LPS, PE18:0/22:6, and PE18:0/22:6_LPS, *n* = 6.

The heatmap with hierarchical clustering analysis (Figure 2) shows the 50 most significantly modulated proteins across the six experimental conditions (Supplementary Table S2), selected based on statistical significance (lowest *p-values*) and categorized according to their Gene Ontology (GO) terms. The analysis revealed a clear separation between controls (CT and CT_LPS) and PE-treated groups (PE18:0/20:4, PE18:0/22:6, PE18:0/20:4_LPS, and PE18:0/22:6_LPS), highlighting the distinct effects of LPS stimulation and PE supplementation on the macrophage proteome.

**Figure 2:**
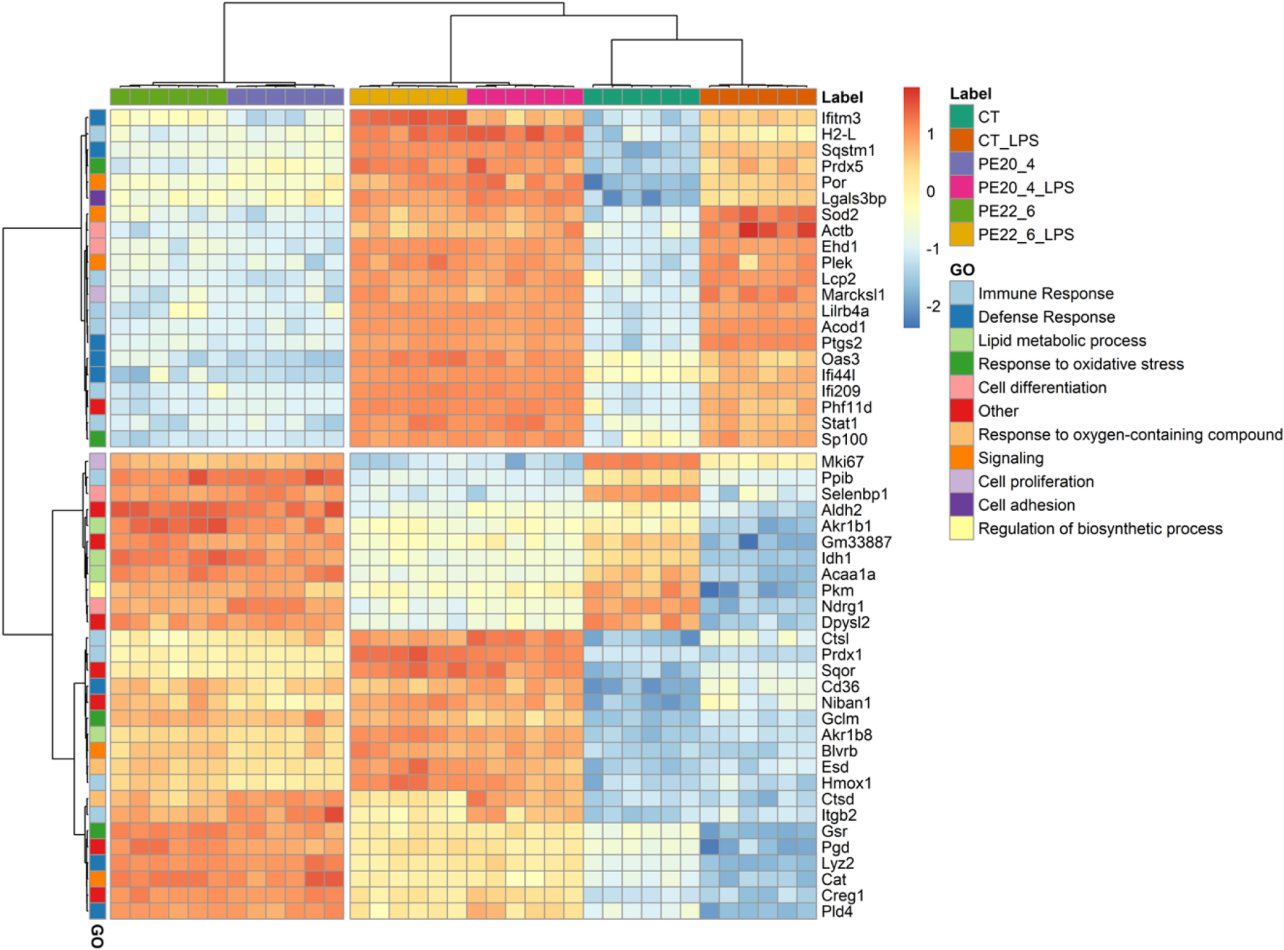
Two-dimensional hierarchical clustering heatmap of the 50 most significantly modulated proteins across the six experimental conditions: CT, CT_LPS, PE18:0/22:6, PE18:0/22:6_LPS, PE18:0/20:4, and PE18:0/20:4_LPS. Proteins are annotated by their associated biological process Gene Ontology (GO) terms. The sample dendrogram (top) represents the similarity among proteomic profiles, while the protein dendrogram (left) clusters proteins by RA patterns, shown as a color gradient from blue (lower RA) to red (higher RA).

LPS stimulation markedly altered the macrophage proteome. In the first protein cluster, 21 proteins were significantly upregulated in CT_LPS compared with CT (*p.adj* < 0.0001), most associated with immune and defense responses (11 proteins). Upregulated proteins included Interferon-induced transmembrane protein 3 (Ifitm3), Superoxide dismutase 2 (Sod2), Prostaglandin G/H synthase 2 (Ptgs2), Interferon-induced protein 44-like (Ifi44l), and Signal transducer and activator of transcription 1 (Stat1). Additional proteins such as Galectin-3-binding protein (Lgals3bp), MARCKS-related protein (Marcksl1), cis-aconitate decarboxylase (Acod1), and Nuclear autoantigen Sp-100 (Sp100) were also upregulated, consistent with an M1-like inflammatory profile previously reported in macrophage polarization studies.^8,32^ Among non-LPS-treated conditions (CT, PE18:0/22:6, and PE18:0/20:4), protein expression patterns within the first cluster were largely similar, indicating minimal basal variability.

In contrast, proteins in the second cluster (Figure 2) showed markedly higher relative abundance (RA) in PE-supplemented macrophages compared with both CT and CT_LPS, indicating that PE treatment remodels the proteome of resting macrophages by enhancing defense and metabolic pathways without inducing a classical pro-inflammatory (M1-like) phenotype. A subset of 11 proteins showed higher abundance in CT compared to CT_LPS (*p.adj* < 0.0001), primarily involved in lipid metabolism, suggesting that LPS activation suppresses lipid metabolic pathways in favor of glycolytic and immune processes. Within this second cluster, antioxidant proteins Peroxiredoxin 1 (Prdx1), Heme oxygenase 1 (Hmox1), Glutathione reductase (Gsr), and Catalase (Cat) were significantly upregulated in both PE18:0/22:6- and PE18:0/20:4-treated macrophages (*p.adj* < 0.0001). Proteins linked to lipid metabolism and immune regulation, including Cluster of Differentiation 36 (CD36), Phospholipase D4 (Pld4), and Lysozyme 2 (Lyz2), were also increased relative to CT and CT_LPS, reflecting metabolic adaptation. No significant differences were observed between the two PE species, suggesting a shared mechanism of action independent of acyl-chain composition.

In LPS-stimulated macrophages (CT_LPS, PE18:0/22:6_LPS, and PE18:0/20:4_LPS), the first cluster, dominated by pro-inflammatory proteins, remained distinct from non-LPS conditions (*p.adj* < 0.0001). Although these proteins were primarily induced by LPS and largely unaffected by PE supplementation, several, including Ifitm3, Sequestosome-1 (Sqstm1), and Peroxiredoxin 5 (Prdx5), displayed higher relative abundance in PE-treated groups compared with CT_LPS (*p.adj* < [0.0001–0.01]).

The second cluster, enriched in lipid metabolism proteins, also showed higher abundance in PE18:0/22:6_LPS and PE18:0/20:4_LPS macrophages relative to CT_LPS (*p.adj* < 0.0001). Notably, a subgroup of ten proteins unrelated to lipid metabolism was specifically upregulated in PE-supplemented, LPS-treated cells. Among these, Prdx1, Hmox1, and CD36 emerged as key mediators of antioxidant and immune defense responses.

### 3.2 Functional and Pathway Reprogramming of Macrophages by PE Species

To further outline the biological processes underlying macrophage reprogramming, GO enrichment analysis was performed on differentially expressed proteins. To characterize the proteomic profiles of M0-like (CT) and M1-like (CT_LPS) macrophages, 179 differentially expressed proteins were identified (*p* < 0.05, |FC| > 1.5) (Figure 3), including 91 upregulated in CT and 88 in CT_LPS. These proteins were mainly associated with immune and defense responses, with multiple overlaps across GO categories, reflecting their participation in interconnected biological processes.

**Figure 3:**
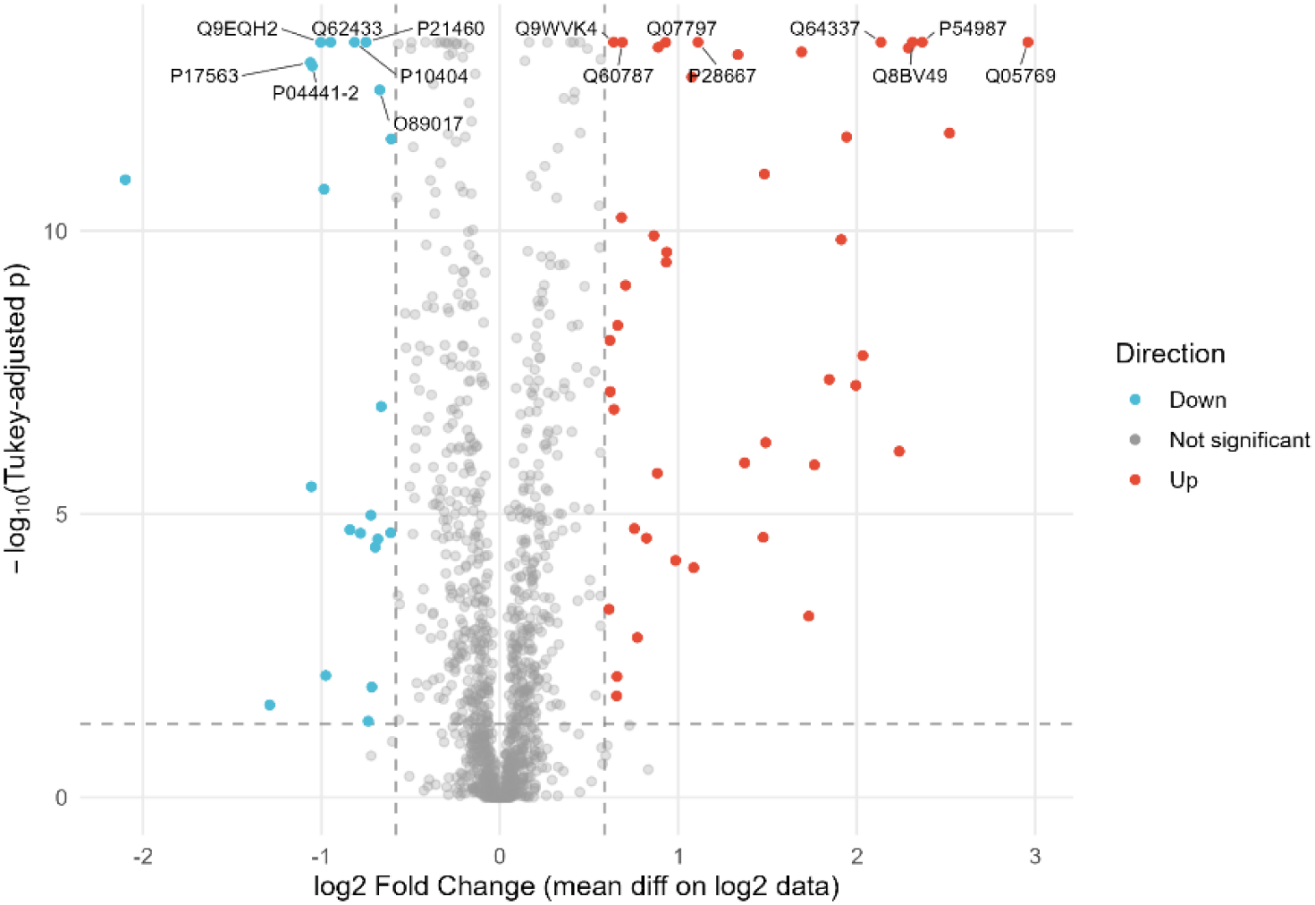
CT_LPS vs CT volcano plot of differentially abundant proteins (−log_10_ Benjamini–Hochberg corrected p-value vs log_2_ fold change CT/CT_LPS). Vertical lines denote ±1.5-fold change; the horizontal line marks significance (*p.adj* = 0.05). Selected prominently regulated proteins are labeled in the plot, upregulated in CT: Erap1 (Q9EQH2), Selenbp1 (P17563), NDRG1 (Q62433), Cd74 (P04441.2), Cst3 (P21460), MLV (P10404), and Lgmn (O89017); Upregulated in CT_LPS: Ehd1 (Q9WVK4), Lcp2 (Q60787), Lgals3bp (Q07797), Marcksl1 (P28667), Sqstsm1 (Q64337), Pyhin1 (Q8BV49), Acod1 (P54987), and Ptgs2 (Q05769). TUKEY | FC threshold = ±0.585­(1.5x), Tukey adj *p* < 0.050 | Significant: 66­(↑44,↓22)|Labels:15.

Proteins upregulated in CT were predominantly linked to lipid metabolic pathways, including fatty-acid (FA) β-oxidation (9 proteins), Lipid catabolic process (12), and FAO (9), suggesting suppression of these pathways in CT_LPS macrophages (Figure 4a ; Supplementary Table S3). Among the proteins enriched in resting M0-like macrophages were CD74 (H-2 class II histocompatibility antigen gamma chain), PKM (Pyruvate kinase M), and MIF (Macrophage migration inhibitory factor), emphasizing their roles in maintaining the basal metabolic phenotype.

**Figure 4:**
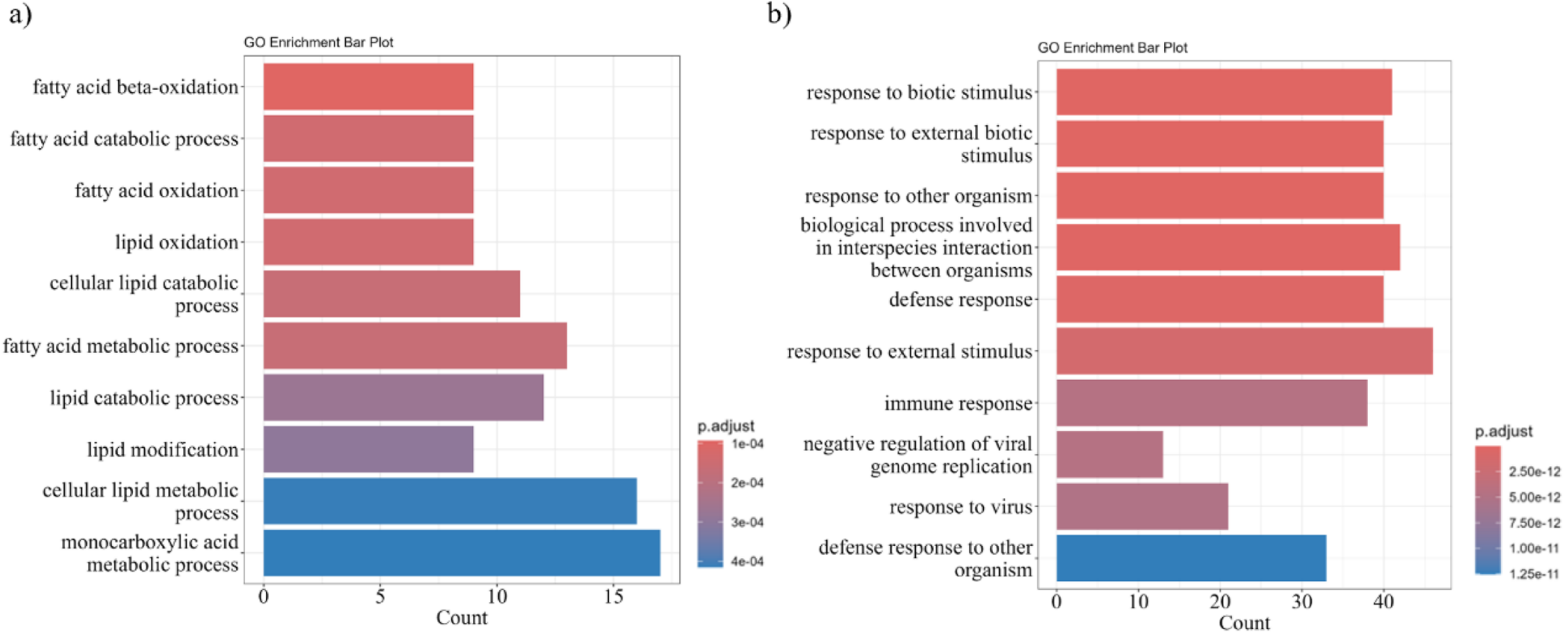
GO enrichment analysis of biological processes: (a) proteins upregulated in CT vs CT_LPS; (b) proteins upregulated in CT_LPS vs CT.

In contrast, CT_LPS macrophages exhibited enrichment in immune-defense proteins, including Ptgs2, Nos2, Nfkb1, Nfkb2, CD40, and CD44 (Figure 4b ; Supplementary Table S4), consistent with M1-like activation and LPS-driven inflammatory pathways. These results delineate the distinct proteomic signatures of M0- and M1-like macrophages: M0-like macrophages display higher lipid-metabolic and FAO-associated proteins, whereas M1-like cells shift toward glycolytic and immunometabolic activity, with elevated expression of pro-inflammatory mediators. This highlights the coupled metabolic and inflammatory reprogramming that accompanies LPS-induced macrophage polarization.

We next compared the proteomes of resting macrophages (CT) and PE18:0/22:6-treated macrophages to assess the effects of phospholipid supplementation. In CT vs PE18:0/22:6-treated cells, 58 proteins were significantly modulated (46 upregulated, 12 downregulated) (Figure 5a). Upregulated proteins were enriched with pathways related to stress response (28 proteins), oxidative stress (11), and inflammation (12) (Figure 6a ; Supplementary Table S5). Key upregulated proteins included Abcc1 (multidrug resistance-associated protein 1), Cat, CD36, Gsr, and Hmox1, all of which are involved in oxidative-stress regulation and inflammation control. Other notable upregulated proteins highlighted in the volcano plot include Gclm (Glutamate-cysteine ligase) and Blvrb (Flavin reductase). Nos2 (Nitric oxide synthase) (*p.adj <* 0.0001) and Nmi (N-myc-interactor) (*p.adj <* 0.001) were among the most significantly downregulated proteins (Figure 5; Supplementary Table S9).

**Figure 5:**
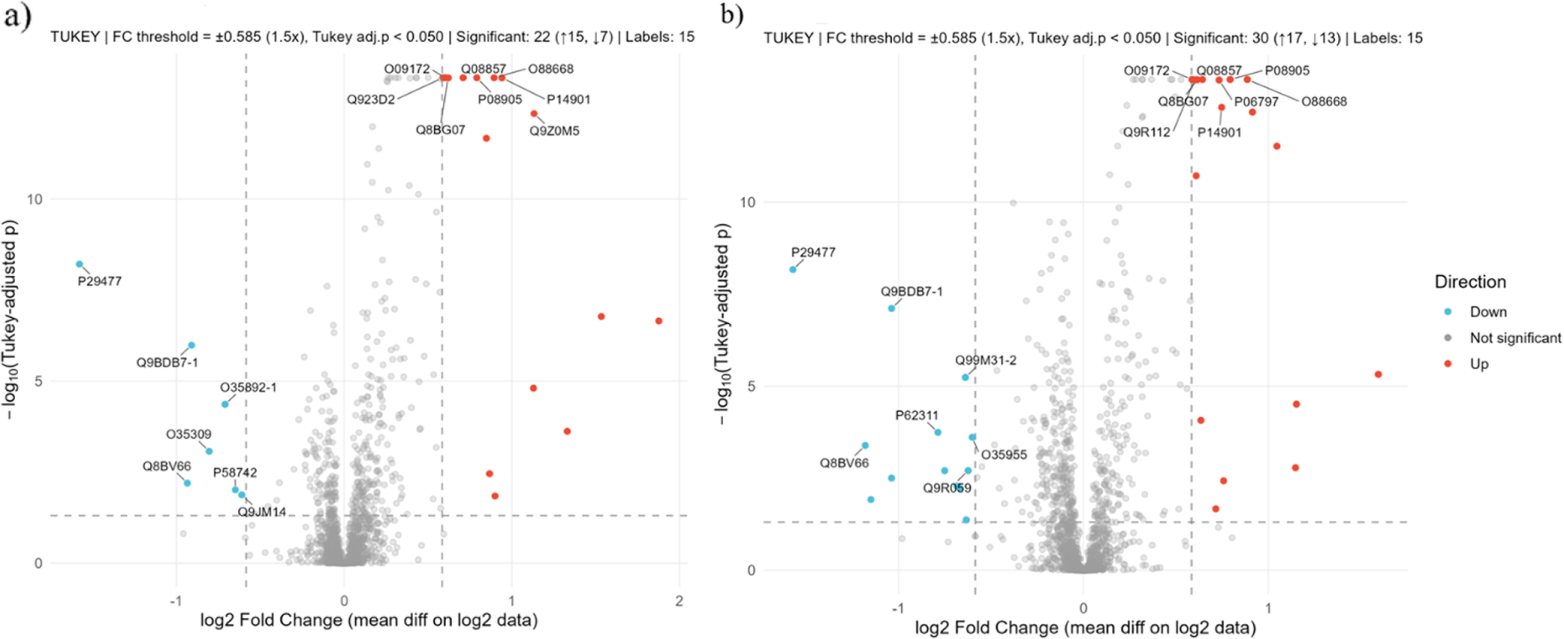
Volcano plot of differentially abundant proteins (−log_10_ Benjamini–Hochberg corrected p-value vs log_2_ fold change). Vertical lines denote ±1.5-fold change; the horizontal line marks significance (*p.adj* = 0.05). Fold change: (a) PE18:0/22:6 vs CT; Upregulated: Lyz2 (P08905), Creg1 (O88668), Gclm (O09172), Sqor (Q9R112), Pld4 (Q8BG07), Ctsl (P06797) and Hmox1 (P14901); Downregulated: Nos2 (P29477), Ifi44l (Q9BDB7.1), Hspa14 (Q99M31.2), Ifi44 (Q8BV66), Lsm3 (P62311), Psmb10 (O35955) and Fhl3 (Q9R059). (b) PE18:0/20:4 vs CT; Upregulated: Gclm (O09172), Blvrb (Q923D2), Pld4 (Q8BG07), Cd36 (Q08857), Lyz2 (P08905), Creg1 (O88668), Hmox1 (P14901) and Lipa (Q9Z0M5); Downregulated: Nos2 (P29477), Ifi44l (Q9BDB7.1), Aaas (P58742), Nt5c (Q9JM14), Ifi44 (Q8BV66), Nmi (O35309) and Sp100 (O35892.1).

**Figure 6:**
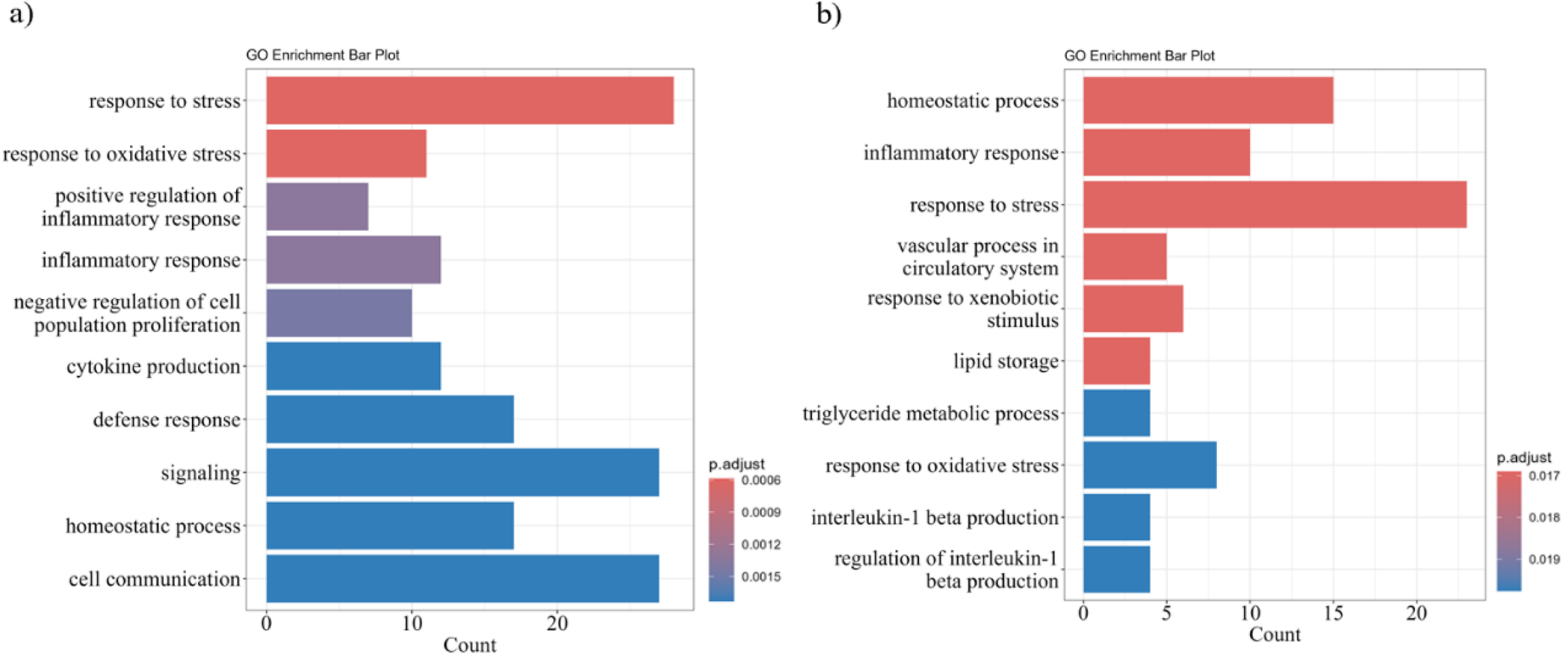
GO enrichment analysis of biological processes for differentially expressed proteins: (a) upregulated in PE18:0/22:6 vs CT; (b) upregulated in PE18:0/20:4 vs CT.

PE18:0/20:4 supplementation significantly altered the abundance of 71 proteins (41 upregulated, 30 downregulated) (Figure 5b). Upregulated proteins were enriched in stress (23), oxidative-stress (8) and inflammatory-response pathways (Figure 6b ; Supplementary Table S6) and associated with homeostasis (15 proteins), lipid storage (4), triglyceride metabolism (4), and IL-1β production (4), indicating strong metabolic and immunomodulatory effects.

Notably, proteins such as Lpl (lipoprotein lipase), CD36, Casp1 (caspase-1), and GSTP1 (glutathione S-transferase P1), all known regulators of IL-1β synthesis, were significantly elevated. In addition, Gclm and Hmox1 were highlighted in the volcano plot as significantly upregulated by PE18:0/20:4 compared with resting macrophages (Figure 5), confirming a shared regulatory effect of PE18:0/22:6 and PE18:0/20:4 on antioxidant defense mechanisms. Among the downregulated proteins (Supplementary Table S10), Nfkb1 and Nfkb2 were identified, suggesting inhibition of NF-κB signaling. Consistently, the concurrent downregulation of Nos2 and Nmi in both PE18:0/22:6 and PE18:0/20:4 treated macrophages further supports attenuation of pro-inflammatory signaling (Supplementary Table S13).

To evaluate how PE18:0/22:6 and PE18:0/20:4 influence macrophage responses under inflammatory conditions, we compared LPS-stimulated macrophages (CT_LPS) with PE-pretreated and LPS-activated cells (PE18:0/22:6_LPS and PE18:0/20:4_LPS). In CT_LPS vs PE18:0/22:6_LPS, 87 proteins were significantly modulated (69 upregulated, 18 downregulated) (Figure 7a). GO analysis revealed enrichment of oxidative-stress response (12 proteins), defense mechanisms (21), catabolic processes (29), and immune regulation (24) (Figure 8a ; Supplementary Table S7). Upregulated proteins (Prdx1, Hmox1, CD36, Abcc1, and GSTP1) mirrored those elevated by PE treatment alone, suggesting consistent antioxidant and detoxification effects. CD74 and Blvrb were also increased in PE18:0/22:6_LPS macrophages relative to CT_LPS (Figure 7). Among the downregulated proteins were Nfkb1 and Nfkb2, consistent with previously reported results under resting conditions (Supplementary Table S11).

**Figure 7:**
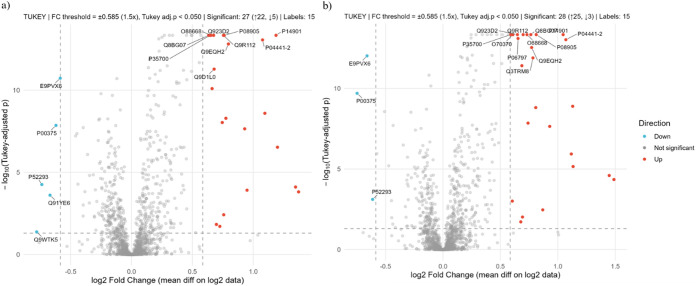
Volcano plot of differentially abundant proteins (−log_10_ Benjamini–Hochberg corrected p-value vs log_2_ fold change). Vertical lines denote ±1.5-fold change; the horizontal line marks significance (*p.adj* = 0.05). Fold change: (a) PE18:0/22:6_LPS vs CT_LPS; Upregulated: Prdx1 (P35700), Hmox1 (P14901), Sqor (Q9R112), Lyz2 (P08905), Pld4 (Q8BG07), Erap1 (Q9EQH2), Creg1 (O88668), Blvrb (Q923D2), Chchd2 (Q9D1L0), Cd74 (P04441.2); Downregulated: Mki67 (E9PVX6), Dhfr (P00375), Kpna2 (P52293), Ipo9 (Q91YE6) and Nfkb2 (Q9WTK5). (b) PE18:0/20:4_LPS vs CT_LPS; Upregulated: Blvrb (Q923D2), Prdx1 (P35700), Ctss (O70370), Erap1 (Q9EQH2), Gvpt (O68668), Sqor (Q9R112), Hk3 (Q3TRM8), and Hmox1 (P14901); Downregulated: Mki67 (E9PVX6), Dhfr (P00375) and Kpna2 (P52293).

**Figure 8:**
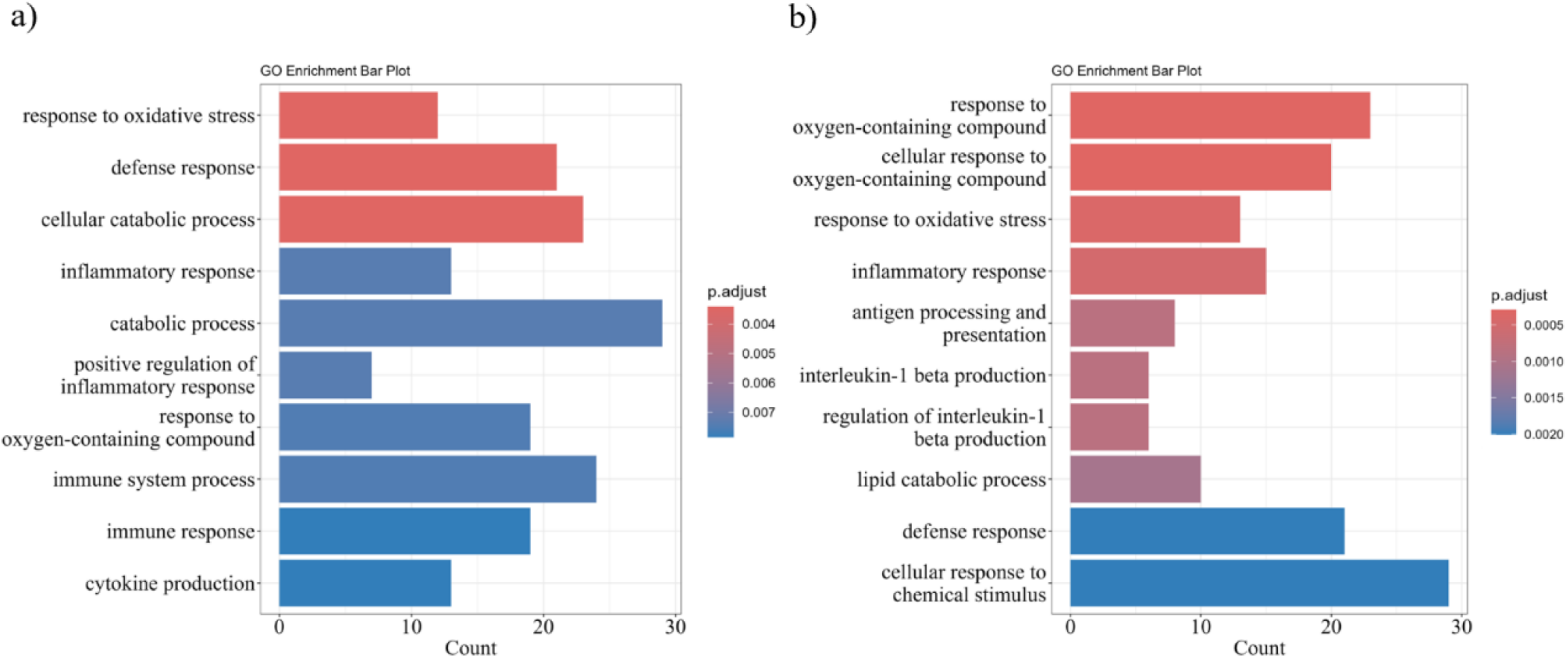
GO enrichment analysis of biological processes for differentially expressed proteins: (a) upregulated in PE18:0/22:6_LPS vs CT_LPS; (b) upregulated in PE18:0/20:4_LPS vs CT_LPS.

In CT_LPS vs PE18:0/20:4_LPS, 93 proteins were differentially expressed (72 upregulated, 21 downregulated) (Figure 7b). Upregulated proteins were associated with oxidative-stress response (13 proteins), inflammation (15), antigen processing (8), and IL-1β production (6) (Figure 8b; Supplementary Table S8). Similar to PE18:0/22:6_LPS, Prdx1, Hmox1, CD36, Abcc1, and GSTP1 were markedly upregulated in PE18:0/20:4_LPS macrophages (Supplementary Table S14). Among the 21 proteins downregulated, Ptgs2 and Nfkb1 significantly decreased compared with CT_LPS (Supplementary Table S12). The reduction of Nfkb1 was consistent across both PE species, whereas Ptgs2 downregulation was specific to PE18:0/20:4_LPS. As these are key inflammatory mediators, their suppression supports an anti-inflammatory action of PE18:0/22:6 and PE18:0/20:4 under LPS stimulation.

### 3.3 Gene Transcription Regulated by PE18:0/20:4 and PE18:0/22:6

To determine whether changes in protein abundance were reflected at the transcriptional level, mRNA expression of key genes was quantified by qPCR (Supplementary Table S15). Targets included inflammatory mediators (*Il1b*, *Ptgs2*), lipid- and adhesion-related receptors (*Cd36*, *Itgb2*), antioxidant enzymes (*Prdx1*, *Hmox1*), the apoptosis-associated regulator *Niban1*, and the lysosomal protease *Catsd*.

LPS stimulation strongly increased *Il1b* (mean log_2_ = 13.56 ± 0.95) and *Ptgs2* (mean log_2_ = 8.15 ± 0.19) transcription, whereas supplementation with PE18:0/22:6 or PE18:0/20:4 markedly reduced mRNA levels of both genes (Figure 9). A comparable decrease in *Ptgs2* expression was observed in PE18:0/22:6_LPS (mean log_2_ = 6.79 ± 0.58) and PE18:0/20:4_LPS (mean log_2_ = 6.70 ± 0.54) macrophages. The effect on *Il1b* was more pronounced for PE18:0/22:6_LPS (mean log_2_ = 10.64 ± 1.27; ∼7.6-fold reduction) than for PE18:0/20:4_LPS (mean log_2_ = 11.62 ± 0.99; ∼3.8-fold reduction) compared with LPS alone, supporting a pro-resolving effect of PE supplementation under inflammatory conditions.

**Figure 9:**
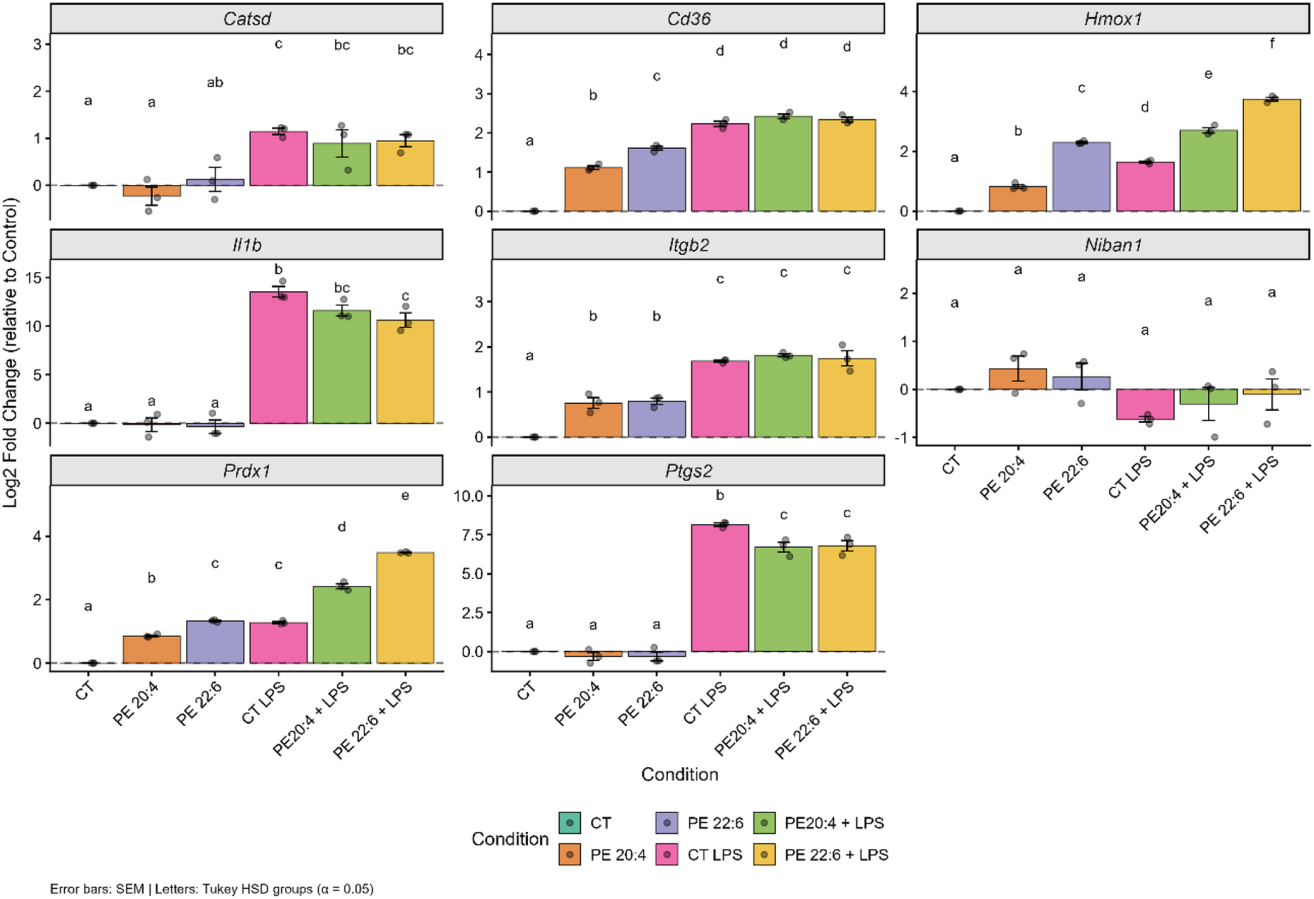
Effect of PE18:0/20:4 and PE18:0/22:6 on gene transcription in RAW 264.7 macrophages. Cells were cultured under control conditions (CT) or treated with PE18:0/20:4 (PE 20:4, 100 μM) or PE18:0/22:6 (PE 22:6, 100 μM) for 24 h, either alone or followed by LPS activation (100 ng/mL) (CT_LPS, PE 20:4_LPS, PE 22:6_LPS). mRNA levels of *Catsd*, *Cd36*, *Hmox1*, *Il1b*, *Itgb2*, *Niban1*, *Prdx1*, and *Ptgs2* are shown as normalized log_2_ fold changes relative to untreated controls (CT), using *Hprt1* as reference. Data represent mean ± SEM from three independent experiments (*n* = 3). Statistical significance was determined by one-way ANOVA with Tukey’s posthoc test, comparing all six experimental conditions; a–f: different letters on top bars indicate significant differences in gene transcription between those groups (*p.adj* < 0.05).

In contrast, antioxidant genes *Hmox1* and *Prdx1* were upregulated in PE-supplemented macrophages under both resting and LPS-stimulated conditions (Figure 9). Under basal conditions, PE18:0/22:6 increased *Hmox1* (mean log_2_ = 2.29 ± 0.05) and *Prdx1* (mean log_2_ = 1.33 ± 0.04), while PE18:0/20:4 induced smaller increases (mean log_2_ = 0.82 ± 0.10 and 0.86 ± 0.05, respectively). Upon LPS activation, this induction was amplified: PE18:0/22:6_LPS macrophages showed ∼4-fold higher *Hmox1* and *Prdx1* transcription (mean log_2_ = 3.74 ± 0.11 and 3.48 ± 0.02), and PE18:0/20:4_LPS cells ∼2-fold (mean log_2_ = 2.70 ± 0.16 and 2.42 ± 0.13) relative to LPS alone, which indicates an enhanced antioxidant defenses following PE treatment.

PE18:0/22:6 and PE18:0/20:4 also increased transcription of adhesion and lipid-uptake genes (Figure 9). Under resting conditions, *Cd36* transcription increased after supplementation, more prominently with PE18:0/22:6 (mean log_2_ = 1.61 ± 0.08) than PE18:0/20:4. Similarly, *Itgb2* expression increased ∼1.7-fold in both PE treatments (mean log_2_ = 0.80 ± 0.12 and 0.75 ± 0.21, respectively). Although these genes remained upregulated following LPS stimulation, differences versus LPS alone were not statistically significant.

*Niban1* mRNA levels increased in resting PE-treated macrophages but were suppressed by LPS alone (Figure 9). PE supplementation partially restored *Niban1* transcription in LPS-treated cells (mean log_2_ = −0.10 ± 0.56 for PE18:0/22:6_LPS and −0.30 ± 0.60 for PE18:0/20:4_LPS, vs −0.62 ± 0.10 for LPS alone). Given its role in cellular stress resistance and apoptosis regulation, *Niban1* upregulation suggests that PE species may enhance macrophage resilience under inflammatory stress.

*Catsd* transcription showed a distinct pattern, decreasing only after PE18:0/20:4 supplementation under basal conditions (mean log_2_ = −0.23 ± 0.34) (Figure 9). A modest reduction was also observed in PE-supplemented, LPS-treated macrophages (*Catsd* mean log_2_ = 0.95 ± 0.22 for PE18:0/22:6_LPS; 0.89 ± 0.50 for PE18:0/20:4_LPS; vs 1.15 ± 0.11 for LPS alone), although these changes were not statistically significant.

### 3.4 Metabolomic Profiling of PE18:0/22:6- and PE18:0/20:4-Treated Macrophages

Untargeted LC-MS-based metabolomics revealed broad metabolic remodeling of macrophages in response to PE18:0/22:6 and PE18:0/20:4 supplementation, with 93 metabolites identified (Supplementary Table S16). Multivariate and hierarchical clustering analyses (Supplementary Figures S1–S2) showed clear separation between LPS-stimulated and nonstimulated macrophages, with additional clustering by PE treatment, indicating that LPS drives the primary metabolic shift while PE induces distinct remodeling under both basal and inflammatory conditions.

Pathway enrichment analysis (Figure 10; Supplementary Table S17) identified histidine and arginine/proline metabolism as the most significantly affected pathways between CT and CT_LPS macrophages (*p* < 0.001; impact > 0.2). l-arginine levels were higher in CT macrophages, while citrulline, putrescine, and N-acetylputrescine increased upon LPS stimulation, consistent with enhanced NO and polyamine synthesis. Histidine metabolism showed decreased histidine and increased histamine in CT_LPS macrophages, reflecting activation of histamine biosynthesis. Taurine metabolism was identified with a pathway impact of 0.8. In LPS-activated macrophages, taurine levels were elevated compared to CT, whereas its precursor hypotaurine was higher in CT than in CT_LPS, reflecting an inflammation-associated shift in amino acid metabolism.

**Figure 10:**
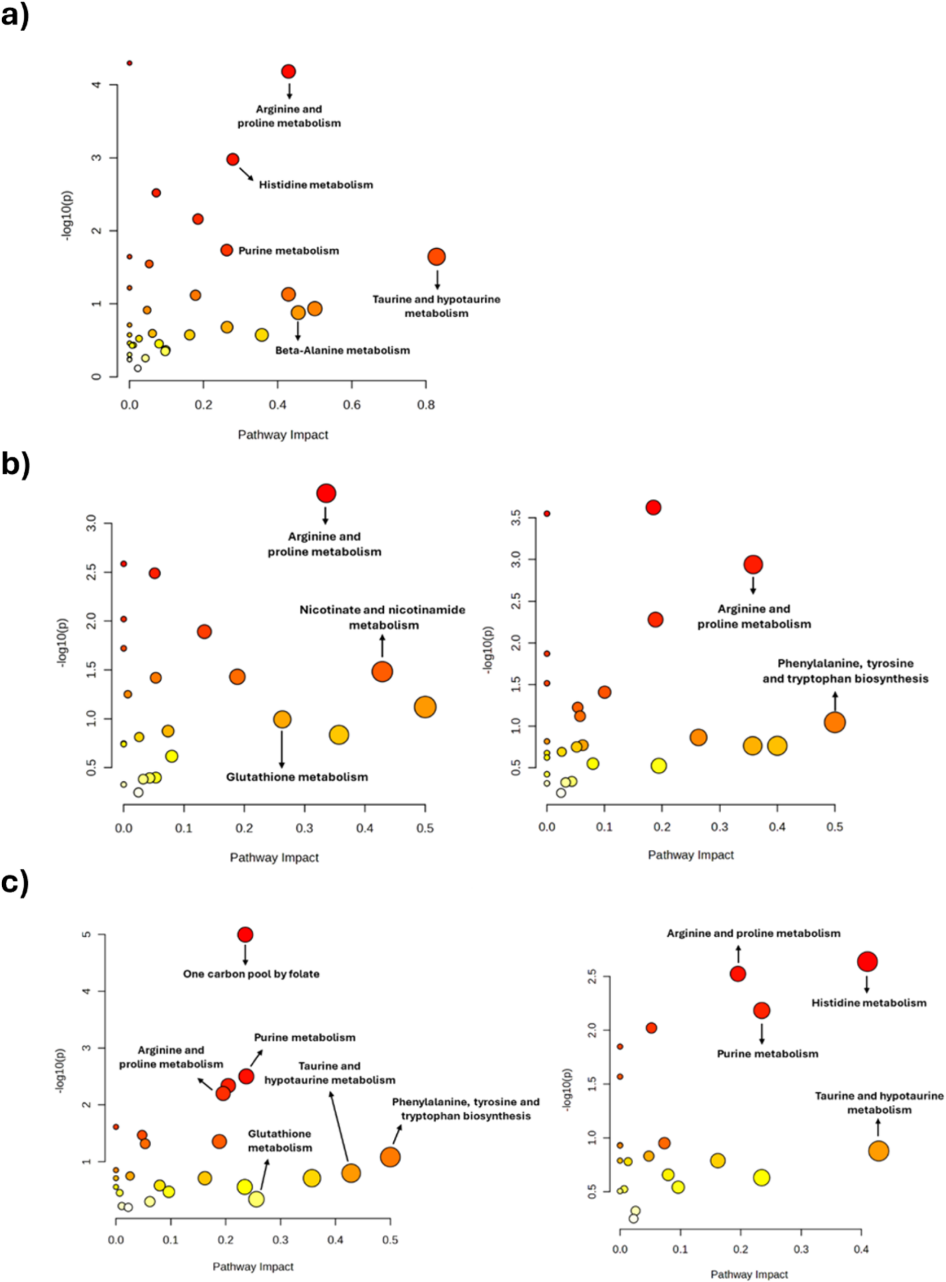
Pathway enrichment analysis of RAW 264.7 macrophage metabolomes a) CT vs CT_LPS; b) CT/PE18:0_22:6 vs CT/PE18:0_20:4; c) CT_LPS/PE18:0_22:6_LPS vs CT_LPS/PE18:0_20:4_LPS. The position along the *x*-axis corresponds to the pathway impact score. The *y*-axis shows the statistical significance (−log_10_(p)), derived from enrichment analysis. Bubble size indicates the number of matched metabolites (Hits), and bubble color reflects *p-value* significance, with deeper red indicating higher enrichment.

In PE18:0/22:6- and PE18:0/20:4-treated macrophages, arginine/proline metabolism remained significantly enriched (*p* < 0.001; impact ≥ 0.2), accompanied by increased l-arginine levels. PE18:0/20:4 macrophages show elevated levels of putrescine and N-acetylputrescine compared to CT. These findings suggest that PE supplementation redirects arginine metabolism from pro-inflammatory NO synthesis toward polyamine biosynthesis, promoting an M2-like anti-inflammatory profile.

Although glutathione metabolism did not reach pathway-level significance, PE18:0/22:6 supplementation markedly elevated glutathione, suggesting enhanced antioxidant capacity (Figure 11).

**Figure 11:**
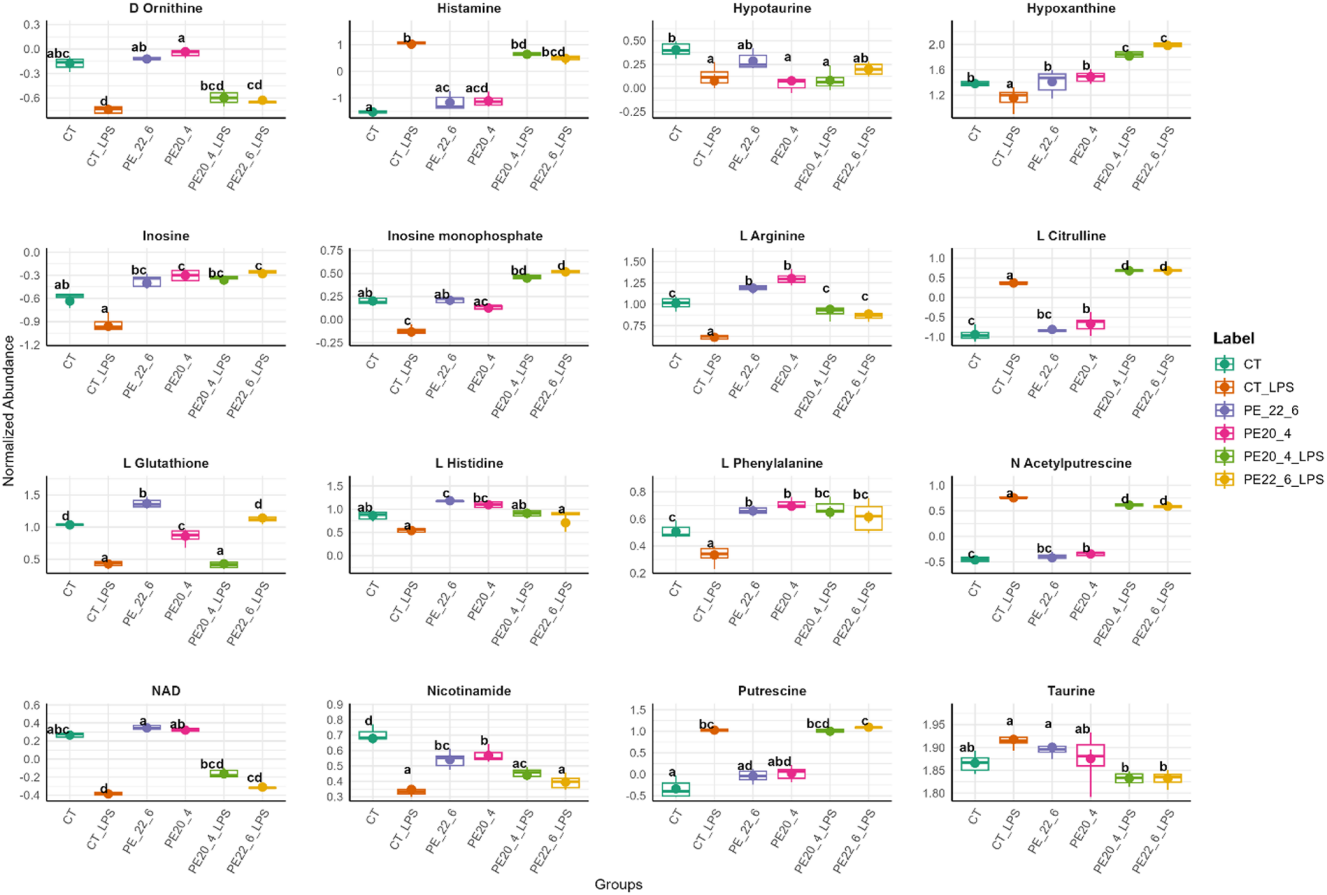
Representative boxplots of metabolites highlighted in pathway analysis. Statistical significance was determined by one-way ANOVA with Tukey’s post hoc test, with all six conditions compared simultaneously; a-d: different letters on boxplots indicate significant differences between the groups (*p.adj* < 0.05).

Upon LPS stimulation, PE treatment significantly modulated several metabolic routes. PE18:0/22:6_LPS macrophages showed enrichment of one-carbon pool by folate, purine, cysteine/methionine, and arginine/proline metabolism (*p* < 0.01; impact ≥ 0.2), whereas PE18:0/20:4_LPS macrophages primarily affected purine, histidine, and arginine/proline metabolism (*p* < 0.01; impact ≥ 0.2). Common pathways in both treatments included purine and arginine/proline metabolism, reflecting shared metabolic reprogramming.

Notably, both PE species restored purine metabolism disrupted by LPS, elevating inosine monophosphate (IMP) and inosine while reversing LPS-induced depletion of hypoxanthine. In addition, PE18:0/22:6_LPS and PE18:0/20:4_LPS macrophages exhibited increased l-arginine and l-citrulline compared to CT_LPS (Figure 11). Conversely, in both PE-treated conditions taurine levels were reduced compared to macrophages stimulated with LPS alone (CT_LPS).

## 4 Discussion

The study of macrophage immunometabolism is fundamental for understanding the mechanisms that drive inflammatory activation and its regulation by bioactive lipid mediators. Here, we investigated the immunomodulatory properties of two native PE, PE18:0/20:4 (AA-containing) and PE18:0/22:6 (DHA-containing), using LC-MS/MS to profile proteomic and metabolomic changes in resting (M0-like) and LPS-activated (M1-like) macrophages 24 h poststimulation. Under analogous conditions, we previously showed efficient incorporation of these species, with 6.5 and 3.1-fold increases in PE18:0/20:4 and PE18:0/22:6, respectively.^19^ Oxidized PE (oxPE) species were detected at much lower levels (≈1000-fold lower for PE18:0/20:4;O vs PE18:0/20:4 and ≈400-fold lower for PE18:0/22:6;O vs PE18:0/22:6), suggesting they represent only a minor fraction of the PE pool under our conditions. While we cannot fully exclude biological contributions from trace oxPE, the magnitude of this difference argues against oxPE being the primary drivers of the effects observed.

LPS induced a pronounced shift from an M0-like to an M1-like profile, with extensive reprogramming of inflammatory and stress-response pathways. Key proteins upregulated in CT_LPS, including Ifitm3, Sod2, Ptgs2, Ifi44l, Stat1, Lgals3bp, Marckl1, Acod1, and Sp100, are consistent with canonical LPS-driven NF-κB and interferon signaling and enhanced eicosanoid metabolism.^8,32,33^ In contrast, proteins enriched in CT were associated with fatty acid β-oxidation and lipid catabolism, reflecting suppression of these pathways upon LPS stimulation and the well-described shift from FAO/OXPHOS toward glycolysis in M1-like macrophages.^1,5,9,34^ Metabolomics further supported this transition, with decreased l-arginine (consistent with iNOS-dependent consumption), increased putrescine and N-acetylputrescine (polyamine pathway activation),^35^ and histidine depletion with histamine accumulation, indicative of histamine-driven immune signaling.^36,37^ Together, these adaptations delineate a robust pro-inflammatory phenotype in CT_LPS relative to CT.

Supplementation with PE18:0/22:6 or PE18:0/20:4 induced a coordinated antioxidant and immunoregulatory phenotype in macrophages that was evident under resting conditions and sustained following LPS-induced inflammatory response. In M0-like macrophages, both PE species increased key oxidative-stress defense proteins (Prdx1, Hmox1, Gsr, Gclm, and Cat), supported by elevated *Hmox*1 and *Prdx1* transcription and enhanced glutathione (GSH) metabolism. Prdx1 and Cat detoxify hydrogen peroxide (H_2_O_2_), thereby protecting against ROS-mediated damage,^38,39^ while Gsr and Gclm sustain intracellular GSH to support ROS neutralization.^40,41^ Hmox1, as a central component of the oxidative stress response, catalyzes heme degradation and mitigates oxidative stress.^42^ PE supplementation also elevated CD36 expression (and *Cd36* transcripts), consistent with altered lipid uptake and metabolic reprogramming.^43,44^ In PE18:0/22:6-treated cells, enrichment analysis highlighted stress-response, and inflammatory pathways, with upregulation of Abcc1 and Gsr, consistent with enhanced glutathione-dependent antioxidant defenses.^45,46^ PE18:0/20:4 supplementation induced a similar enrichment profile in stress-response, including IL-1β regulation, underscoring an immunoregulatory role for this PE species. Although PE18:0/20:4 modulated several IL-1β regulatory proteins (Lpl, CD36, CASP1, and GSTP1),^47,48^ *Il1b* transcription remained unchanged in resting macrophages, suggesting a net inhibitory influence under basal conditions. Consistently, both PE species reduced Nos2 and Nmi expression, indicating suppression of canonical inflammatory pathways,^49–51^ with PE18:0/20:4 additionally decreasing Nfkb1 and Nfkb2. This coordinated reduction suggests dampening of NF-κB-dependent inflammatory cascades and reduced NO production,^52^ as reported for PUFA-containing PL in RAW 264.7 macrophages.^17,53^ This anti-inflammatory shift was reinforced by arginine remodeling, whereas M1-like macrophages convert arginine to NO via iNOS, M2-like channel it through arginase-1 (ARG1) to ornithine and polyamines.^54,55^ PE supplementation increased intracellular l-arginine while suppressing Nos2 expression,^19^ consistent with reduced iNOS-induced NO synthesis.^54,55^

Having delineated the LPS-induced M1-like baseline and the remodeling elicited by PE18:0/22:6 and PE18:0/20:4, we next address how prior supplementation with these lipids shaped the macrophage response to LPS. PE18:0/22:6_LPS and PE18:0/20:4_LPS macrophages exhibited sustained upregulation of antioxidant and stress-response proteins, including Sqstm1, Hmox1, Prdx1 and Prdx5, together with increased *Hmox1* and *Prdx1* transcription. Induction of Sqstm1, together with reduced taurine levels, a known activator of Nrf2,^56,57^ is consistent with activation of the Keap1-Nrf2 positive feedback loop under inflammatory conditions.^58,59^ Activation of Keap1-Nrf2 drives the expression of antioxidant response elements (ARE)-dependent transcription of cytoprotective genes,^60,61^ providing a plausible mechanism for the observed upregulation of Hmox1, Gsr, and Cat in PE18:0/22:6_LPS and PE18:0/20:4_LPS macrophages. This could occur indirectly through mild oxidative/electrophilic stress that alter Keap1 cysteine reactivity, and/or via increased Sqstm1/p62 abundance, which is known to modulate Nrf2 signaling through a positive feedback loop.^62^ In our proteomics data set, Sqstm1/p62 increased modestly with PE alone and more strongly in the PE + LPS conditions compared with LPS alone. These observations are compatible with Nrf2/ARE pathway involvement, but they do not demonstrate causality. Further experiments are required to determine the mechanism underlying PE-induced antioxidant gene expression.^61–63^

Conceptually, our findings align with prior work showing that OxPL, reprogram macrophage phenotypes toward redox/stress-adaptive states (e.g., Mox-like phenotype) characterized by Nrf2-linked antioxidant gene expression and metabolic remodeling.^64–66^ OxPL have been described as bioactive mediators with DAMP-like properties, promoting Nrf2-driven antioxidant defenses,^65^ stress-response pathways, and metabolic reprogramming via TLR2-Syk signaling.^64^ In contrast, the present study examines native PUFA-containing PE species (PE18:0/20:4 and PE18:0/22:6), which in our assays elicit a redox-adaptive and inflammation-restrained profile at baseline and during LPS challenge, without evidence that they behave as canonical OxPL-like danger signals. GO analysis of PE18:0/22:6_LPS and PE18:0/20:4_LPS upregulated proteins reinforced oxidative-stress and detoxification pathways, with Abcc1 and GSTP1 prominently elevated (mirroring their induction in resting PE-treated macrophages). Additionally, in PE18:0/20:4_LPS, enriched pathways included antigen processing/presentation and IL-1β production, as observed in undifferentiated cells. Both PE18:0/20:4 and PE18:0/22:6 supplemented before LPS, reduced *Il1b* transcription relative to LPS alone, indicating a pro-resolving influence. Metabolomics concurred this effect, with increased inosine levels by both PE18:0/22:6 and PE18:0/20:4, a metabolite linked to diminished IL-1β responses in LPS-activated macrophages.^67,68^ Among downregulated proteins, Nfkb1 and Nfkb2 decreased in PE18:0/22:6_LPS, while Ptgs2 and Nfkb1 were reduced in PE18:0/20:4_LPS versus CT_LPS. The consistent reduction of Nfkb1 suggests that, despite LPS-driven NF-κB activation, PE can mitigate this axis by lowering transcription factor abundance. These results align with prior observations that EPA-PE inhibits NF-κB activation in RAW 264.7 macrophages,^17^ raising the possibility that the ethanolamine headgroup contributes to this effect.

Because we used LPS as the sole inflammatory trigger, our conclusions are restricted specifically to LPS/TLR4-mediated activation. PE enrichment may influence plasma-membrane organization and thereby modulate assembly/activation of the TLR4 signaling complex and downstream inflammatory nodes such as p38 MAPK and NF-κB.^69^ These possibilities remain hypothetical and require direct validation with alternative stimuli, such as TNF-α or IL-1β. Consistently, PE18:0/22:6 and PE18:0/20:4 also attenuated LPS-induced *Ptgs2* transcription, reinforcing their immunoregulatory action on cytokine/eicosanoid pathways.

Collectively, these data demonstrate that PE18:0/22:6 and PE18:0/20:4 promote a macrophage state associated with enhanced antioxidant defenses, attenuated LPS-induced inflammatory signaling, and metabolic features consistent with a more immunoregulatory phenotype. This phenotype is evident under resting conditions and persists during LPS challenge, distinguishing PE-treated macrophages from both M0-like cells and LPS-driven M1-like activation states. This supports a role for these PE species in shaping redox-resilient, inflammation-controlled macrophage responses in the context of LPS/TLR4-mediated activation. Mechanistically, PE enrichment could influence the efficiency of LPS sensing and signaling by modulating plasma-membrane organization and/or the assembly and activation of the CD14-TLR4 signaling complex.^70^ In addition, by analogy to observations reported for other membrane phospholipids,^71,72^ PE enrichment may indirectly constrain downstream inflammatory nodes such as p38 MAPK and NF-κB, though direct evidence remains to be established.

## 5 Conclusion

Phospholipids are key regulators of immune metabolism, yet their specific influence on macrophage function remains incompletely defined. Here, we show that PE species with distinct acyl-chains reprogram macrophage immunometabolism in a context-dependent manner. PE supplementation at rest did not elicit a classical pro-inflammatory profile but remodeled protein expression toward increased antioxidant defenses and CD36 upregulation, consistent with a redox-fortified, metabolically adapted state with dampened canonical inflammatory mediators. Under LPS stimulation, prior PE exposure enhanced antioxidant responses and attenuated inflammatory signaling, with increased abundance of proteins involved in redox control and lipid metabolism. Consistently, qPCR confirmed reduced *Il1b* and *Ptgs2* expression together with increased *Prdx1*, *Hmox1*, and *Cd36*. Metabolomics converged with these findings, indicating reinforced glutathione/redox pathways (more prominent with PE18:0/22:6), a redirection of arginine toward polyamines, and context-dependent shifts in purine and amino-acid metabolism. Overall, our findings position native PUFA-containing PE species as modulators of macrophage immunometabolism that restrain excessive NF-κB/eicosanoid signaling and enhance antioxidant capacity. Future work should evaluate pathway causality (e.g., Keap1-Nrf2 dependence, CD36 involvement), dissect the relative contributions of the PE headgroup and its acyl chains, and validate these effects in primary macrophages and *in vivo* models, toward PE-based strategies that modify inflammatory responses while preserving host defense.

## Supplementary Material





## Data Availability

The mass spectrometry proteomics data have been deposited to the ProteomeXchange Consortium via the PRIDE^73^ partner repository with the data set identifier PXD069938 and 10.6019/PXD069938. The metabolomics data have been deposited to MetaboLights^74^ repository with the study identifier MTBLS13235. All other relevant data are provided in the Supporting Information.

## Notes

(XLSX). The Supporting Information is available free of charge at https://pubs.acs.org/doi/10.1021/acs.jproteome.5c01131.

Table S1: oligonucleotide primer pairs used for qPCR analysis; Table S2: univariate analysis was performed to determine the 50 most significantly modulated proteins across the 6 experimental conditions; Table S3: GO enrichment analysis of biological processes: proteins upregulated in CT vs CT_LPS; Table S4: GO enrichment analysis of biological processes: proteins upregulated in CT_LPS vs CT; Table S5: GO enrichment analysis of biological processes: proteins upregulated in PE18:0/22:6 compared to CT; Table S6: GO enrichment analysis of biological processes: proteins upregulated in PE18:0/20:4 compared to CT; Table S7: GO enrichment analysis of biological processes: proteins upregulated in PE18:0/22:6_LPS compared to CT_LPS; Table S8: GO enrichment analysis of biological processes: upregulated in PE18:0/20:4_LPS compared to CT_LPS; Table S9: downregulated proteins in PE18:0/22:6 compared to CT; Table S10: downregulated proteins in PE18:0/20:4 compared to CT_LPS; Table S11: downregulated proteins in PE18:0/22:6_LPS compared to CT_LPS; Table S12: downregulated proteins in PE18:0/20:4_LPS compared to CT_LPS; Table S13: proteins commonly upregulated and downregulated between PE18:0/20:4 and PE18:0/22:6; Table S14: proteins upregulated in PE18:0/22:6_LPS and PE18:0/20:4_LPS; Table S15: effect of PE18:0/20:4 and PE18:0/22:6 on gene transcription; Table S16: metabolite species identified by LC-MS in RAW 264.7 macrophages supplemented with PE18:0/22:6 and PE18:0/20:4 alone and in the presence of LPS; Table 17: metabolite pathway enrichment analysis performed using MetaboAnalyst 6.0 pathway analysis module (XLSX)
Figure S1: principal component analysis (PCA) score plot of the metabolite data set; Figure S2: hierarchical clustering analysis of the 50 most significant metabolites across the 6 experimental conditions (PDF)
