# Supplementary material for: Integrated Proteomics and Metabolomics Analyses Reveal That Phosphatidylethanolamine Reprograms Macrophage Immunometabolism and Attenuates LPS-Driven Inflammation
Source: J Proteome Res. 2026 Apr 8;25(5):2437–51. doi: 10.1021/acs.jproteome.5c01131 (PMC13140139; doi:10.1021/acs.jproteome.5c01131)
Supplement: Supplementary file 2 [file pr5c01131_si_002.pdf]

Integrated Proteomics and Metabolomics Analyses Reveal that Phosphatidylethanolamine Reprograms Macrophage Immunometabolism and Attenuates LPS-Driven Inflammation

Tatiana Maurício [1,2,3], Bruno Neves [3], M. Rosário Domingues [1,2], Pedro Domingues[1*]

[1] Mass Spectrometry Centre, LAQV-REQUIMTE, Department of Chemistry, University of Aveiro, Santiago University Campus, 3810-193 Aveiro, Portugal

[2] CESAM - Centre for Environmental and Marine Studies, Department of Chemistry, University of Aveiro, Santiago University Campus, 3810-193 Aveiro, Portugal

[3] Department of Medical Sciences and Institute of Biomedicine, iBiMED, University of Aveiro, 3810-193 Aveiro, Portugal

***Corresponding author:** Pedro Domingues,

Department of Chemistry, University of Aveiro, Santiago University Campus, 3810-193 Aveiro, Portugal

e-mail: p.domingues@ua.pt.

List of contents:

Supplementary Table S1. Oligonucleotide primer pairs used for qPCR analysis. (XLSX)

Supplementary Table S2. Univariate analysis was performed to determine the 50 most significantly modulated proteins across the 6 experimental conditions. These proteins were selected based on their statistical significance and p-values are listed accordingly. (n = 6) (* if q < 0.05, ** if q < 0.01, *** if q < 0.001 and if **** q < 0.0001). (XLSX)

Supplementary Table S3. GO enrichment analysis of biological processes: proteins upregulated in CT vs CT_LPS. (XLSX)

Supplementary Table S4. GO enrichment analysis of biological processes: proteins upregulated in CT_LPS vs CT. (XLSX)

Supplementary Table S5. GO enrichment analysis of biological processes: proteins upregulated in PE 18:0/22:6 compared to CT. (XLSX)

Supplementary Table S6. GO enrichment analysis of biological processes: proteins upregulated in PE 18:0/20:4 compared to CT. (XLSX)

Supplementary Table S7. GO enrichment analysis of biological processes: proteins upregulated in PE 18:0/22:6_LPS compared to CT_LPS. (XLSX)

Supplementary Table S8. GO enrichment analysis of biological processes: Upregulated in PE 18:0/20:4_LPS compared to CT_LPS. (XLSX)

Supplementary Table S9. Downregulated proteins in PE18:0/22:6 compared to CT. (XLSX)

Supplementary Table S10. Downregulated proteins in PE18:0/20:4 compared to CT_LPS. (XLSX)

Supplementary Table S11. Downregulated proteins in PE18:0/22:6_LPS compared to CT_LPS. (XLSX)

Supplementary Table S12. Downregulated proteins in PE18:0/20:4_LPS compared to CT_LPS. (XLSX)

Supplementary Table S13. Proteins commonly upregulated (green) and downregulated (red) between PE18:0/20:4 and PE18:0/22:6 associated with response to stress (GO:0006950). (XLSX)

Supplementary Table S14. Proteins upregulated in PE18:0/22:6_LPS and PE18:0/20:4_LPS. Common proteins upregulated are highlighted in bold red. (XLSX)

Supplementary Table S15. Effect of PE18:0/20:4 and PE18:0/22:6 on gene transcription. mRNA levels of Il1b, Cd36, Hmox1, Prdx1, Ptgs2 Catsd, Itgb2, and Niban1, are shown as normalized $\log_2$ fold changes relative to untreated controls (CT), using Hprt1 as reference. Data represent mean ± SD from three independent experiments (n = 3). (XLSX)

Supplementary Table S16. Metabolite species identified by LC-MS in RAW 264.7 macrophages supplemented with PE18:0/22:6 and PE18:0/20:4 alone and in the presence of LPS. (XLSX)

Supplementary Table 17. Metabolite pathway enrichment analysis performed using MetaboAnalyst 6.0 pathway analysis module. The table lists only significant enriched metabolic pathways identified in pairwise comparisons between control groups (CT and CT_LPS) and PE treatment groups. (XLSX)

Supplementary Figure S1. Principal component analysis (PCA) score plot of the metabolite dataset: control (CT), control with LPS (CT_LPS), PE18:0/20:4 (abbreviated as PE_20_4), PE18:0/20:4_LPS (abbreviated as PE_20_4_LPS), PE18:0/22:6 (abbreviated as PE_22_6), and PE18:0/22:6_LPS (abbreviated as PE_22_6_LPS), n = 6. (DOCX)

Supplementary Figure S2. Hierarchical clustering analysis of the 50 most significant metabolites across the 6 experimental conditions: control (CT), control with LPS (CT_LPS), PE18:0/20:4 (abbreviated as PE_20_4), PE18:0/20:4_LPS (abbreviated as PE_20_4_LPS), PE18:0/22:6 (abbreviated as PE_22_6), and PE18:0/22:6_LPS (abbreviated as PE_22_6_LPS), n = 6. (DOCX)

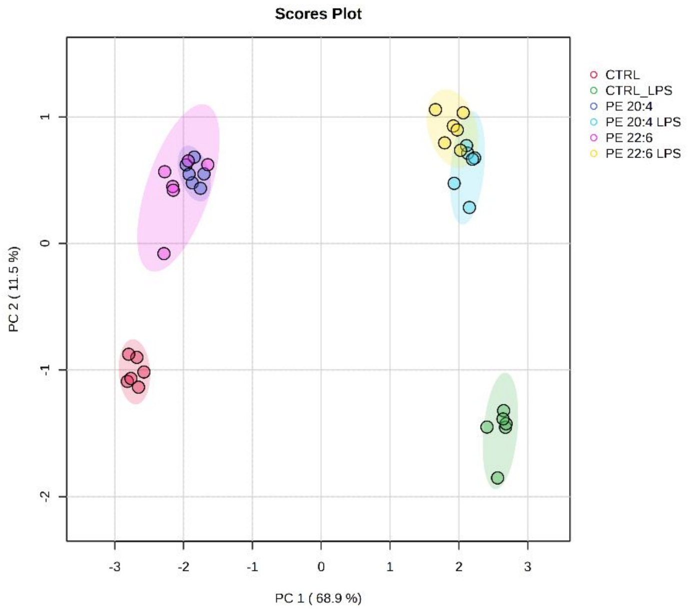

Supplementary Figure S1. Principal component analysis (PCA) score plot of the metabolite dataset: control (CT), control with LPS (CT_LPS), PE18:0/20:4 (abbreviated as PE_20_4), PE18:0/20:4_LPS (abbreviated as PE_20_4_LPS), PE18:0/22:6 (abbreviated as PE_22_6), and PE18:0/22:6_LPS (abbreviated as PE_22_6_LPS), n = 6.

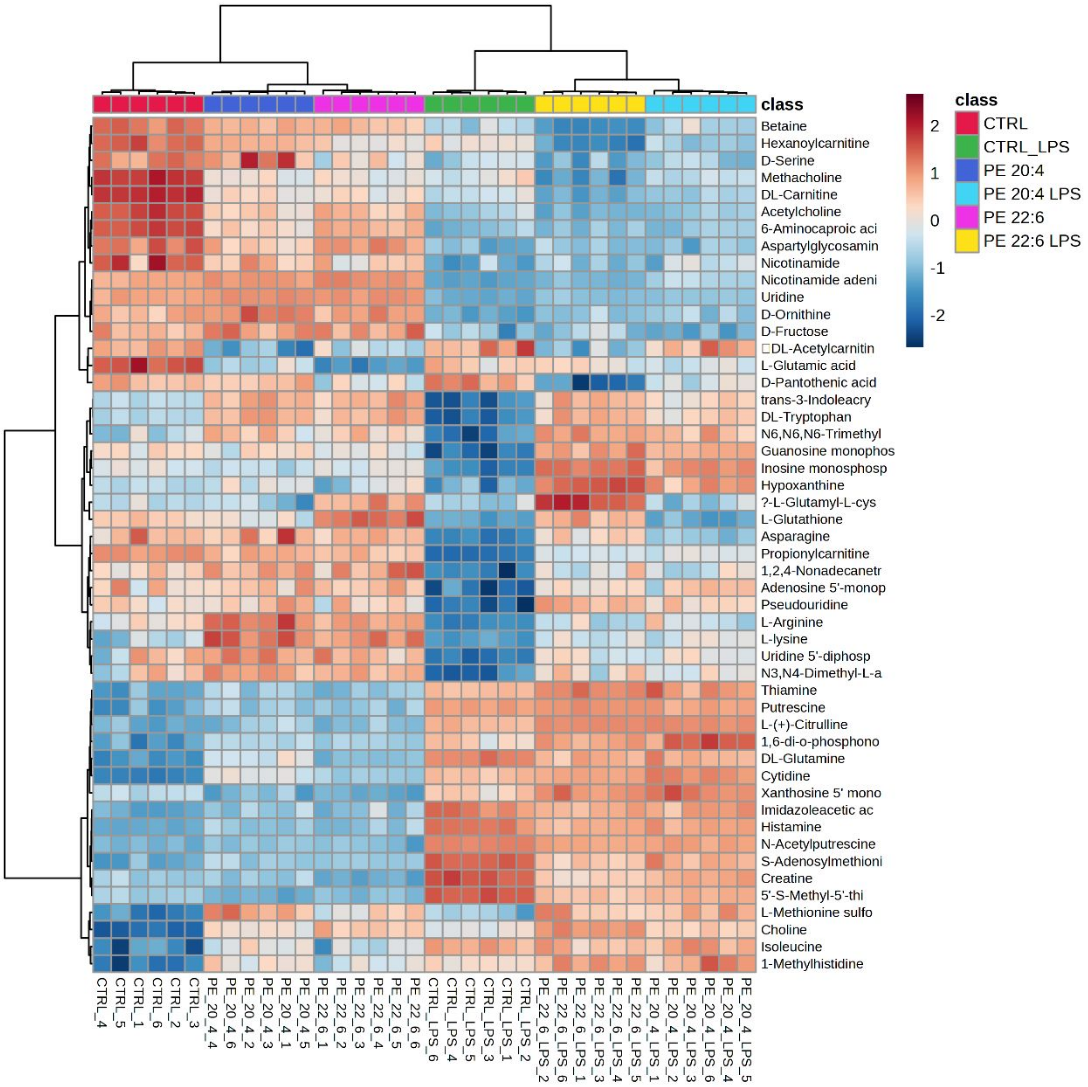

Supplementary Figure S2. Hierarchical clustering analysis of the 50 most significant metabolites across the 6 experimental conditions: control (CT), control with LPS (CT_LPS), PE18:0/20:4 (abbreviated as PE_20_4), PE18:0/20:4_LPS (abbreviated as PE_20_4_LPS), PE18:0/22:6 (abbreviated as PE_22_6), and PE18:0/22:6_LPS (abbreviated as PE_22_6_LPS), n = 6.
